# Peritoneal Metastases from Breast Cancer: A Scoping Review

**DOI:** 10.7759/cureus.5367

**Published:** 2019-08-11

**Authors:** Michèle Beniey

**Affiliations:** 1 Surgery / General Surgery, University of Montreal, Montreal, CAN

**Keywords:** cytoreductive surgery, hyperthermic intraperitoneal chemotherapy (hipec), peritoneal metastases, breast cancer

## Abstract

Breast cancer is the leading cause of cancer deaths amongst American women aged 20 to 59. While the incidence of breast cancer has been increasing, its mortality rates have significantly declined from 1989 to 2016. As a result, the number of survivors considerably increased. This impacts the detection and management of recurrences. Peritoneal metastases from breast cancer is a rare and challenging clinical presentation. There is a lack of knowledge syntheses and specific recommendations for the management of breast cancer peritoneal metastases. This review aims to determine the pattern of spread, prognosis, diagnosis, and role of surgery in this subset of patients.

Relevant studies were searched in PubMed and Web of Science between April and June 2019. Included studies were written in English and reported data on breast cancer peritoneal or gastrointestinal metastases. Articles published before 1990, case reports, editorials, and articles with no full text available were excluded. Data abstraction was performed for citation information, population, sample, methods, relevant results, mentioned limitations, and study design.

The search identified 505 unique reports. A total of 21 articles were included in the synthesis. Sixteen articles were observational studies, four were experimental, and one article was a proof-of-concept study. Amongst all observational studies, the diagnostic methods and criteria for breast cancer carcinomatosis were particularly heterogeneous, including ascites cytology, biopsy, surgical exploration, and various computed tomography (CT) findings. The majority of pathology and imaging reports demonstrated that breast cancer peritoneal metastases are mainly associated with invasive lobular carcinoma (ILC) and the following intrinsic subtypes: HER2-enriched, luminal B and basal-like. Experimental studies demonstrated that peritoneal metastases can be studied using breast cancer xenograft models. Somatic loss of both p53 and E-cadherin was associated with ILC peritoneal spread. Studies on prognosis and treatment highlighted that peritoneal metastases were associated with a poorer prognosis than other metastatic sites. In terms of surgical management, there is a paucity of data on the outcomes of hyperthermic intraperitoneal chemotherapy (HIPEC) in these patients. However, included studies suggested a role for cytoreductive surgery in selected patients when there is no residual disease after the procedure.

This review summarizes data on the development, diagnosis, prognosis, and treatment of breast cancer peritoneal and gastrointestinal metastases. Patients’ survival is significantly reduced in comparison with other distant metastatic sites. A deeper understanding of the invasion mechanisms and the role of surgery will be important.

## Introduction and background

One in eight American women develop breast cancer during their lifetime [1]. According to 2019 cancer statistics, breast cancer accounts for about 30% of all new cancer cases [1]. While breast cancer mortality rates have considerably decreased over the past years, incidence rates have increased by 0.4% per year between 2006 and 2015 [1]. However, the breast cancer five-year relative survival rates were amongst the highest (90%) of all cancer types diagnosed between 2008 and 2014 [1]. This is the result of a reduction in smoking and reflects progress in the detection of early-stage breast cancer [1]. With the aging of the population and the increase in the proportion of breast cancer survivors, it will be important to promptly recognize and treat distant recurrences.

Although the recognition and treatment of breast cancer metastases to sites such as bone, liver, lungs, and brain are well-documented, breast cancer spread to peritoneal surfaces is a poorly defined entity. Breast cancer carcinomatosis is a rare clinical presentation that usually occurs during a progression event or can be detected on initial diagnosis in some cases [2-4]. Even though peritoneal and gastrointestinal metastases of breast cancer represent a clinical challenge, there is a lack of data in the literature and reported data are particularly scattered. The purpose of this scoping review was to determine the pattern of spread, diagnostic methods, and prognosis of breast cancer gastrointestinal and peritoneal metastases. In addition, this review aimed to assess the role of cytoreductive surgery with or without hyperthermic intraperitoneal chemotherapy (HIPEC) in affected patients.

## Review

Search strategy

Two databases were searched: PubMed and Web of Science. The search strategy is described in the Appendix. Articles published before 1990 were excluded since breast cancer diagnosis and treatment have considerably evolved, and the methodology of studies has significantly improved ever since. Each search strategy was designed to have the most appropriate results possible and is tailored to the associated database. The search was conducted between April 6, 2019, and June 24, 2019.

Inclusion and exclusion criteria

**Figure 1:**
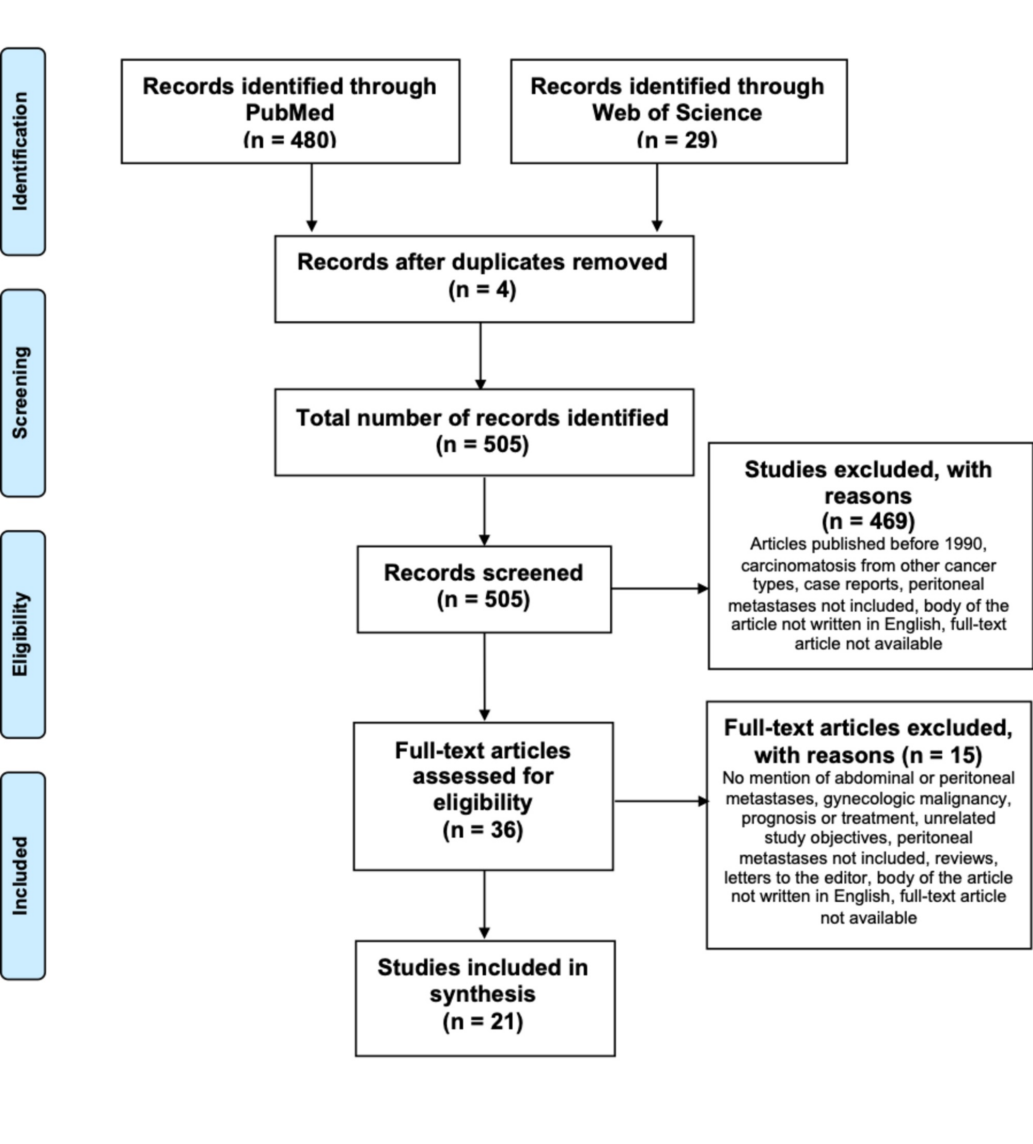
Prisma flow diagram

Articles included in this review were English-written and reported data on the pattern of spread, diagnosis, or treatment of peritoneal or gastrointestinal metastases from breast cancer (Figure 1). Case reports, editorials, and reviews were excluded, as well as articles with unavailable full text and studies published before January 1990.

Data extraction

One author screened all titles and abstracts and selected all included articles. A two-stage approach was used. In the first stage, abstracts and titles were screened to broadly identify all reports related to peritoneal or gastrointestinal spread from breast cancer. Then, full-text articles were assessed for inclusion. Data abstraction was performed for citation information, population, sample, methods, relevant results, mentioned limitations, and study design. A summary of the main characteristics of all included studies is reported in Table 1. Descriptive statistics with articles' publication year and place were undertaken in GraphPad Prism (version 7.00 pour Mac OS X, GraphPad Software, La Jolla California USA).

**Table 1:** Characteristics of included studies and main objectives CEA: carcinoembryogenic antigen; HIPEC: hyperthermic intraperitoneal chemotherapy; ILC: invasive lobular carcinoma; IDC: invasive ductal carcinoma; MMP: matrix metalloproteinase; USA: United States of America [5-25]

Article	Design	Objectives	Total N	Methods	Mentioned limitations
Pattern of spread (N=10)					
Lamovec, 1991 (Slovenia)	Cohort study	Difference in metastatic sites between ILC and IDC	226	Retrospective review of autopsy records and histology	None
Antic, 2010 (USA)	Cohort study	Tumor type and single-cell pattern of peritoneal effusions in metastatic breast cancer	819	Retrospective review of patients’ pathologic reports and clinical files	None
Jain, 1993 (USA)	Cohort study	Differences in metastatic sites between ILC and IDC	1391	Retrospective review of clinical records	None
Inoue, 2017 (Japan)	Cohort study	Clinical significance of peritoneal metastases from breast cancer	330	Retrospective review of clinical records	None
Winston, 2000 (USA)	Cohort study	Pattern of spread of metastatic ILC to the chest, abdomen and pelvis	57	Retrospective review of CT images	No direct comparison with IDC; CT is not the gold standard for diagnosis; Biopsy-proven metastases not performed in all patients.
DiPiro, 2019 (USA)	Cohort study	Frequency, patterns and prognosis of ILC with abdominal metastases	116	Retrospective analysis of CT images	Retrospective design; Lack of generalizability (tertiary center); Variability in timing and number of images between patients; No cross-checking.
Kennecke, 2010 (Canada)	Experimental, correlational	Correlations between breast cancer molecular subtypes and distant metastatic site	3726	Tissue microarray and immunohistochemical analyses	Changes in adjuvant therapy guidelines over time; Overestimation of the risk of relapse;
Mitra, 2006 (USA)	Experimental	Role of intrinsic FAK (Focal Adhesion Kinase) activity in promoting tumor progression	24	Whole-body fluorescent microscopy in orthotopic breast cancer xenografts	None
Derksen, 2011 (Netherlands)	Experimental	Development of a preclinical model for breast carcinomatosis	86	Transgenic mice model and orthotopic breast cancer xenografts	None
Diagnosis (N=2)					
Noh, 2012 (Korea)	Experimental	Diagnostic precision of MMP-2 and MMP-9 in ascites and pleural effusions in metastatic breast cancer	36	Chemiluminescent enzyme immunoassay on body fluids for CEA detection; Zymography and ELISA for matrix metalloproteinase	Patients heterogeneity; Retrospective study; Small sample size; Arbitrarily defined cut-off levels.
De Mattos-Arruda, 2014 (USA)	Proof-of-concept study	Use of targeted massively parallel sequencing to determine the origin of abdominal metastases	1	Targeted capture massively parallel sequencing	Only one patient; Lack of generalizability due to BRCA1 mutation.
Prognosis and treatment (N=10)					
Bertozzi, 2015 (Italy)	Cohort study	Prognosis of breast cancer patients with peritoneal carcinomatosis compared to other metastatic sites	289	Retrospective patients’ charts review	None
Flanagan, 2018 (Ireland)	Cohort study	Prognosis of patients with peritoneal metastases from extra-abdominal primary tumors	543	Retrospective patients’ charts review	Missed cases due to under-reporting and due to the inclusion of hospitalized patients only.
Tuthill, 2009 (United Kingdom)	Cohort study	Management and prognosis of patients with peritoneal metastases from breast cancer	44	Retrospective patients’ charts review	Small sample size; Heterogeneity in treatment modalities.
Abu-Rustum, 1997 (USA)	Cohort study	Outcomes of surgical management in patients with breast cancer carcinomatosis	40	Retrospective patients’ charts review	None
Eitan, 2003 (USA)	Cohort study	Role of surgical resection in patients breast cancer metastases to the abdomen and pelvis	59	Retrospective patients’ charts review	None
Garg, 2005 (USA)	Cohort study	Etiology, predictive features of peritoneal carcinomatosis and prognosis of cytoreduction	79	Retrospective patients’ charts review	Selection bias; Incomplete clinical data due to the retrospective design; Evolution in treatment over time; Completeness of surgical resection is subjective.
Cardi, 2015 (Italy)	Cohort study	Cytoreduction and HIPEC in patients with peritoneal metastases from rare primary tumors	27	Retrospective patients’ charts review	None
Cardi, 2013 (Italy)	Case series	Outcomes of HIPEC in peritoneal metastases	5	Retrospective patients’ charts review	None
McLemore, 2005 (USA)	Cohort study	Treatment outcomes in patients with gastrointestinal and peritoneal metastases from breast cancer	73	Retrospective patients’ charts review	Article only includes women with pathology proven metastases.
Gusani, 2008 (USA)	Cohort study	Morbidity and mortality of HIPEC and cytoreduction in peritoneal carcinomatosis from different cancer types	122	Retrospective patients’ charts review	None

Study selection

**Figure 2:**
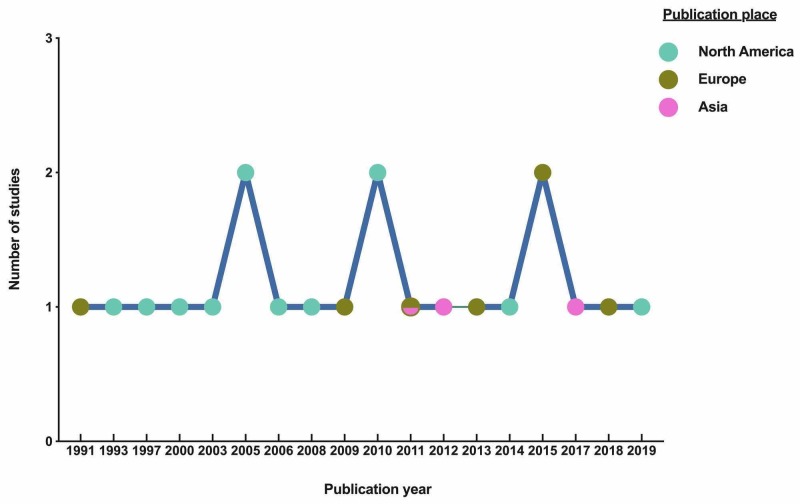
Number of included studies per year and places of publication

**Figure 3:**
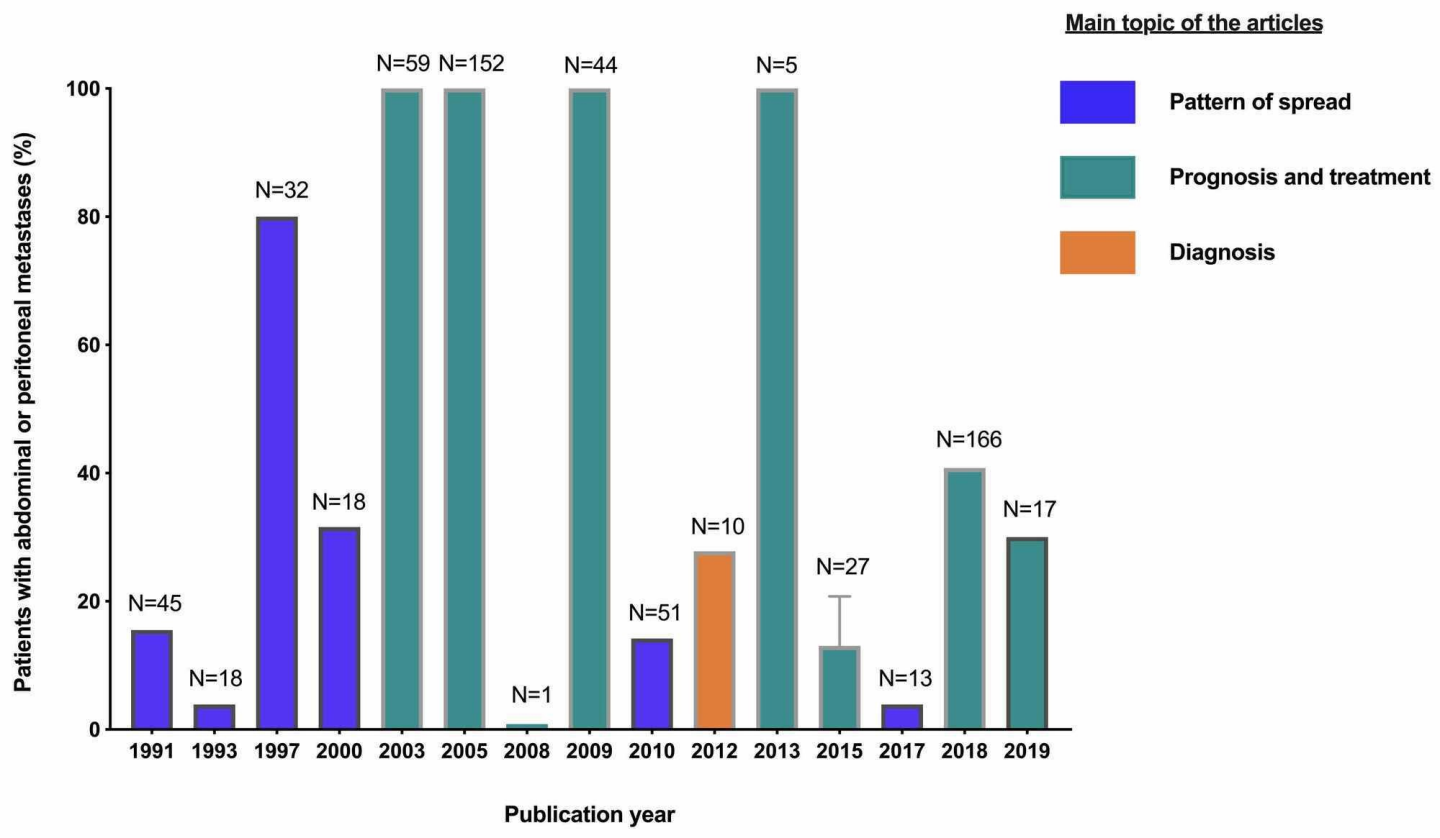
Proportion of patients with peritoneal or gastrointestinal metastases in the included studies Proof-of-concept study and experimental studies were excluded.

A total of 505 unique articles were identified by the search. The number of identified and excluded articles at each stage is described in Figure 1. Breast cancer carcinomatosis is under-reported in the literature. A maximum of two articles per year was eligible for inclusion despite our broad inclusion criteria and most authors were in North America (Figure 2). In addition, only four studies have focused on breast cancer carcinomatosis with all included patients having peritoneal or gastrointestinal spread from breast cancer (Figure 3). In most studies, breast cancer carcinomatosis was only present and evaluated in less than 50% of patients (Figure 3).

Further, the definition and diagnostic criteria of peritoneal carcinomatosis were heterogeneous amongst included studies (Table 2).

**Table 2:** Clinical data used to establish the diagnosis of peritoneal metastases in included cohort studies In studies with several diagnostic criteria, the criteria were used alternatively. [5-11], [16-25]

Author	Pathology			Abdominal computed tomography criteria				Clinical file
	Biopsy	Cytology	Surgery	Not specified	Ascites	Peritoneal lesion	Omental lesion	
Lamovec J et al.	X							
Antic T et al.		X						
Jain S et al.		X	X	X				
Inoue M et al.				X				X
Winston C B et al.						X		
DiPiro P J et al.	X					X	X	
Kennecke H et al.					X	X	X	
Bertozzi S et al.		X		X				
Flanagan M et al.		X		X				X
Tuthill M et al.				X				
Abu-Rustum NR et al.			X					
Eitan R et al.			X					
Garg R et al.			X					
Cardi M et al.			X					
McLemore EC et al.	X			X				
Gusani NJ et al.	X		X	X				

Pattern of spread

Several groups have studied the difference in metastatic spread between invasive lobular carcinoma (ILC) and invasive ductal carcinoma (IDC) and found statistically significant differences in the proportion of patients with peritoneal metastases. Most comparative studies demonstrated that invasive lobular carcinoma had a greater propensity to spread to peritoneal surfaces [5,7-8].

Two groups have assessed autopsy and pathology records to evaluate the prevalence of metastases [5-6]. In one study, autopsy records and hematoxylin and eosin slides from 195 patients with invasive ductal carcinoma and 25 patients with ILC were reviewed [5]. Sixty percent of patients with lobular carcinoma had peritoneal metastases with a diffuse macroscopic pattern (i.e., multiple small nodules measuring 2 mm to 3 mm in diameter). The proportion of peritoneal metastases from IDC was 15.4% and most depicted a nodular pattern. Peritoneal metastases were also detected in three of four cases of mixed IDC-ILC. In another study, peritoneal effusions with positive cytology for breast carcinoma from 51 patients were identified [6]. About 63% of the samples originated from IDC compared to 21.6% from ILC and 9.8% from mixed carcinoma [6]. These findings contrast with the above-mentioned studies. However, the diagnosis of peritoneal carcinoma cannot be made solely on ascites cytology. In fact, the presence of positive cytology may be the result of liver metastases. Furthermore, on microscopy, a diffuse single-cell pattern was typically seen in ILC, similar to mesothelial cells, whereas most cases of IDC displayed a clustering pattern [6]. The single-cell pattern was characterized by nucleus eccentricity, plasmacytoid features, and secretory vacuoles [6].

Other groups have focused on clinical records. Compared to 3% of patients diagnosed with IDC, up to 11% of ILC patients had peritoneal metastases diagnosed with CT and operative reports (P = 0.006) [7]. Autopsy data examination in a subgroup of patients revealed that three of four cases of ILC developed peritoneal metastases compared to three of 52 IDC cases [7]. Similarly, Inoue et al. reported that 68.8% of lobular carcinomas had spread to the peritoneum compared to 1% of ductal carcinomas [8].

Retrospective analysis of CT images was performed by radiologists in two studies to determine the pattern of spread of ILC to the abdomen and pelvis [9-10]. In one study, gastrointestinal metastases (i.e., small bowel, colon, or stomach), and peritoneal metastases were present in 32% and 30% of patients, respectively [9]. Histology analysis confirmed the diagnosis of metastases in 44 patients, including 11% of patients with peritoneal and mesentery spread and 18% of gastrointestinal metastases [9]. In the second study, the peritoneum was the second most common site of metastases (23%) initially, and the most frequent (47%) relapse site during a median follow-up period of 146 months [10]. The most frequent diagnostic finding was diffuse peritoneal thickening and 49 of 51 patients with ascites also had peritoneal involvement on CT.

This scoping review also includes three experimental studies aiming to identify correlations between the development of peritoneal metastases and different breast cancer subtypes, as well as to elucidate molecular mechanisms behind the development of peritoneal metastases. Kennecke et al. assessed correlations between breast cancer intrinsic subtypes [26-27] and sites of metastatic disease using a tissue microarray of 3726 tumors [11]. Clinical charts were reviewed to assess the presence of peritoneal involvement. Both pleural and peritoneal metastatic sites were pooled together and analyzed as one distant metastatic site (n=423). HER2-enriched tumors had the highest 15-year site-specific cumulative incidence of pleural and peritoneal metastases (16.2% to 16%), followed by luminal B (14.7%) and basal-like tumors (12.8%). [11]. Luminal A tumors had the lowest cumulative incidence (7.8%) [11]. These results were statistically significant (P < 0.001) [11] and are congruent with the prognostic significance of the intrinsic subtypes. Indeed, basal and HER2-enriched tumors have a lower survival rate while luminal B tumors are more aggressive than luminal A [26-27]. Nevertheless, a second analysis was performed after excluding patients with no recurrence and no statistically significant differences were detected [11]. As for the proportion of pleural and peritoneal metastases amongst all patients with distant metastases, the difference across the intrinsic subtypes was less pronounced. Luminal B (35.2%) and HER2-enriched tumors (31.6% to 34.2%) had the highest incidence of pleural and peritoneal metastases, followed by basal-like tumors (29.6%) and luminal A tumors (28.2%) [11]. Together, these results demonstrated that the incidence of peritoneal and gastrointestinal metastases is increased in ILC. However, an important consideration is that subclinical metastases can be missed on CT images and the diagnosis criteria of peritoneal involvement were heterogeneous amongst studies (Table 2).

Peritoneal metastases have been also studied in animal models [12-13]. The inhibition of the expression of focal adhesion kinase (FAK), an intracellular tyrosine kinase involved in cell motility and proliferation, was evaluated [12]. Short hairpin RNA (shRNA) was used to inhibit FAK expression in 4T1 breast cancer cells [12]. 4T1 cells were injected in the mammary fat pad of immunosuppressed mice to create 12 orthotopic breast cancer xenografts [12]. Twelve distinct xenografts were used as controls. Green fluorescent protein immunofluorescence was used to detect cells expressing FAK shRNA [12]. Upon necropsy, the inhibition of FAK expression resulted in local tissue destruction and peritoneal invasion [12]. Interestingly, contrary to controls, only a few GFP-positive tumor implants were detected on the peritoneum and no cells were on the colon [12]. Overall, the inhibition of FAK expression resulted in a lower metastatic burden and the results suggested that FAK promotes the development of peritoneal metastases in breast cancer [12]. However, local tissue destruction and direct peritoneal invasion from the mammary gland to the peritoneum is not a plausible mechanism in humans.

In a separate study, somatic loss of E-cadherin and p53 in the mammary fat pad was established in a transgenic mouse model [13]. In comparison with the loss of p53 alone, the loss of both p53 and E-cadherin resulted in the development of invasive mammary tumors displaying phenotypic similarities with human pleomorphic lobular carcinoma [13]. Diffuse dissemination of discohesive tumor cells was found in the peritoneal cavity, with an invasion pattern similar to the one of human ILC [13]. Tumor cells from the transgenic mice were transduced with luciferase-encoding lentiviruses and orthotopically injected in immunosuppressed mice [13]. Bioluminescence imaging permitted to evaluate distant metastases growth. Tumors harboring a somatic loss of both p53 and E-cadherin developed distant metastases at various sites, including the peritoneum [13].

Diagnosis

Only a few studies have reported using diagnostic methods for breast cancer peritoneal spread. The diagnostic role of matrix metalloproteinase (MMP)-2 and MMP-9 in ascites has been investigated in metastatic breast cancer [14]. All patients (n=7) had positive results for MMP-2 [14]. Compared to pleural effusions, MMP-9 levels in ascites were decreased. Carcinoembryonic antigen (CEA) was also assessed and detected levels were increased compared to pleural effusions [14]. However, the true sensitivity and specificity of these biomarkers cannot be accurately determined in this study due to a lack of pathological confirmation of metastatic disease. Only patients’ clinical information and imaging reports were used to establish the diagnosis of peritoneal metastases. Diagnostic criteria were not reported.

In particular cases, the detection of peritoneal carcinomatosis can precede the one of the primary disease. De Mattos-Arruda et al. reported using massively parallel sequencing in a case of synchronous breast and ovarian malignancy in a BRCA1-mutated patient [15]. The patient was diagnosed with IDC and had a pelvic and an adnexal mass, peritoneal implants, and ascites [15]. The biopsy results of the pelvic and peritoneal nodules were inconclusive [15]. Sequencing was performed on DNA extracted from each lesion and on peripheral blood leukocytes [15]. The results indicated that the lesions were from two distinct primary tumors with breast cancer cells harboring a TP53 frameshift mutation and the ovarian lesion displaying a distinct TP53 nonsense mutation [15]. Further, the peritoneal lesions harbored the same mutation as the ovarian mass, in addition to a distinct Nuclear Receptor Coactivator 2 (NCOA2) mutation [15]. The copy number aberrations were similar between the ovarian mass and the peritoneal nodules. Together, these results indicated that the peritoneal implants originated from an ovarian malignancy [15]. Nevertheless, immunohistochemical analysis for estrogen receptors, progesterone receptors, and human epidermal growth factor receptor 2 (HER2) on distant metastases demonstrated diagnostic accuracy in occult lesions [28] and are now recommended.

Prognosis and treatment

Amongst all the different breast cancer distant metastatic sites, peritoneal metastases were associated with a considerably lower survival rate. Bertozzi et al. looked at risk factors and prognosis in 22 patients with breast cancer and peritoneal metastases [16]. A lower body mass index (BMI), ILC, estrogen receptor expression, extracapsular invasion of nodal metastases, T4 breast cancer, N3 stage, and grade 2 tumors significantly correlated with the presence of peritoneal metastases [16]. A multivariate analysis identified high tumor grade, ILC, and loco-regional involvement as predictors of peritoneal metastases [16]. About 82% of patients with peritoneal disease also had other metastatic sites involved [16]. Patients with peritoneal metastases had the worst overall survival. In another study, aiming to assess the survival rate of patients with peritoneal metastases from extra-abdominal tumors, breast cancer accounted for 40.8% (222/543) of primary tumors [17]. One hundred and seventy-one patients received systemic chemotherapy or antihormonal treatment. Overall survival from the diagnosis of metastases was 5.8 months in patients with peritoneal metastases as compared to 22.6 months in metastatic breast cancer patients with no peritoneal involvement [17]. Patients with metachronous metastases had significantly poorer survival than patients with synchronous metastases [17]. In a retrospective cohort of 44 patients with breast cancer and peritoneal carcinomatosis, 56% of patients received chemotherapy and 14.2% were treated with anti-hormonal therapy [18]. The median survival from the diagnosis of metastatic breast cancer was 20.5 months [18].

In order to establish the role of surgery in this subset of patients, some groups conducted retrospective cohort studies to assess the oncological outcomes of cytoreduction and HIPEC. Amongst 40 patients with recurrent metastatic breast cancer to the abdomen and pelvis, 90% were managed operatively [19]. In seven patients, the surgery consisted of abdominopelvic implants biopsy [19]. At the end of the intervention, 18 of 37 patients with residual disease had macroscopically detectable tumors at the end of the intervention [19]. Overall survival was 24.1 months after a median follow-up of 14.2 months [19]. Despite a 2.5-fold difference, there was no statistically significant difference between the overall survival of patients with no gross residual disease after cytoreductive surgery (4.6 months) and one of the patients with residual disease equal or above 2 cm. However, this could be explained by the small sample size. In addition, the administration of adjuvant chemotherapy and endocrine therapy was associated with improved survival.

Similarly, in a distinct cohort, patients with no residual disease after cytoreductive surgery had an overall survival of 54 months (median follow-up = 21 months) and patients with macroscopic residual disease had an overall survival of 21 months [20]. Interestingly, patients diagnosed with abdominal metastases within five years of initial diagnosis had a significantly poorer prognosis than patients with a recurrence after five years [20]. Garg et al. have drawn similar conclusions in a smaller cohort of 19 patients with recurrent breast cancer [21]. These results demonstrate a role for cytoreductive surgery in metastatic breast cancer to the abdomen when cytoreduction is performed optimally and particularly in patients with peritoneal disease recurring within five years of initial diagnosis. However, all of these studies have only included patients with recurrent breast cancer; most studies on the surgical management of peritoneal metastases on initial diagnosis are case reports. Moreover, there is a lack of data on breast cancer-specific survival.

All included studies evaluating outcomes of both cytoreduction and HIPEC are retrospective, and most reports have a small sample size [22-23,25]. Cardi et al. evaluated the outcomes of HIPEC in 28 patients with peritoneal carcinomatosis from secondary tumors [22]. Patients with extra-abdominal metastases, poor performance status, and severe medical conditions were excluded. Five patients had peritoneal metastases from breast cancer and the median elapsed time between the diagnosis of breast cancer and peritoneal disease was 18 years [22-23]. Mean peritoneal carcinomatosis index (PCI) was 20.2 [29]. Four of the five patients were free of disease, yet the duration of the follow-up period was not specified [22]. The overall survival was 56 months, calculated from cytoreductive surgery and HIPEC. In a larger cohort, 23 patients had gastrointestinal metastases (mostly colorectal) from breast cancer, 32 patients had carcinomatosis, and 18 had both [24]. Twelve patients had gastrointestinal or peritoneal carcinomatosis at the time of first breast cancer diagnosis [24]. Most patients received systemic therapy after the diagnosis of abdominal metastases with palliative surgery performed in 64% of cases (47/73) [24]. Ten patients underwent surgical debulking. Patients with gastric metastases had the worst overall survival [24]. Further, in the presence of gastrointestinal metastases, palliative surgery resulted in increased overall survival (44 months versus nine months, *P* = 0.1) [24].

Main findings of the included studies are summarized in Table 3.

**Table 3:** Summary of main findings ILC: invasive lobular carcinoma; HIPEC: hyperthermic intraperitoneal chemotherapy

Key findings
ILC, high tumor grade and loco-regional involvement are associated with peritoneal metastases.
HER2-enriched, luminal B and basal-like tumors have a greater propensity to spread to the peritoneum.
Findings in othotopic xenografts suggest a role for somatic loss of p53 and E-cadherin in the development of breast cancer peritoneal metastases.
There is variability in the definition and diagnostic criteria used for breast cancer peritoneal metastases including the presence of ascites, positive ascites cytology and peritoneal lesions on CT.
Studies evaluating the role of surgery are mainly small and retrospective.
Cytoreduction and HIPEC demonstrated encouraging results in small cohorts. Larger and more robust studies are needed in order to determine their impact on breast cancer-specific survival.
Studies suggest a role for palliative cytoreductive surgery in selected patients when there is minimal or no residual disease.

## Conclusions

This scoping review provides an overview of the data published since 1990 on the pattern of spread, diagnosis, prognosis, and surgical management of peritoneal metastases from breast cancer. Larger cohorts will be needed to assess the clinical and pathological predictive markers of carcinomatosis development in these patients. Further, breast cancer xenografts are preclinical models that can be used as useful tools for a deeper understanding of the invasion mechanism. Cytoreduction and HIPEC are not the treatment of choice due to the tumor burden and several metastatic sites in most patients. However, this review demonstrates that robust data on the surgical treatment of these patients are lacking. Surgery should be further explored in prospective studies in patients with a low metastatic burden, no extra-abdominal metastases, and a high probability of complete cytoreduction.

## Appendices

**Table 4:** PubMed and Web of Science search strategies

Targeted records				Concept			
Database	Year	Type	Language	Breast cancer	Metastases	Treatment	Restriction
PubMed	All	All	All	Breast cancer; OR breast carcinoma.	Peritoneal metastases	None	None
Web of Science	1990–2019	Articles	English	Breast cancer; OR breast carcinoma.	Peritoneal metastases; OR carcinomatosis; OR peritoneal spread; OR abdominal metastases.	Hyperthermic intraperitoneal chemotherapy; OR HIPEC; OR debulking; OR cytoreductive surgery.	NOT children
